# Diet and macronutrient niche of Asiatic black bear (*Ursus thibetanus*) in two regions of Nepal during summer and autumn

**DOI:** 10.1002/ece3.4926

**Published:** 2019-03-08

**Authors:** Saroj Panthi, Achyut Aryal, Sean C. P. Coogan

**Affiliations:** ^1^ Department of Forests and Soil Conservation Ministry of Forests and Environment Kathmandu Nepal; ^2^ Institute of Natural and Mathematical Sciences Massey University Auckland New Zealand; ^3^ Charles Perkins Centre, School of Life and Environmental Sciences, Faculty of Science The University of Sydney Sydney New South Wales Australia; ^4^ Department of Renewable Resources University of Alberta Edmonton Alberta Canada

**Keywords:** Asiatic black bear, diet, macronutrients, niche breadth, nutritional ecology, *Ursus thibetanus*

## Abstract

Relatively little is known about the nutritional ecology of omnivorous Asiatic black bears (*Ursus thibetanus*) in Nepal. We characterized the diet of black bears in two seasons (June–July, “summer”; and October–November “autumn”) and two study areas (Dhorpatan Hunting Reserve [DHR]; and Kailash Sacred Landscape [KSL]). We then conducted nutritional analysis of species consumed by black bears in each study area, in combination with nutritional estimates from the literature, to estimate the proportions of macronutrients (i.e., protein [P], lipid [L], and carbohydrate [C]) in the seasonal bear foods and diets, as well as their macronutrient niche breadth. We found that bamboo (*Arundinaria* spp.) had the highest relative frequency in both study areas and seasons. Ants and termites were found in DHR diets, but not KSL diets. One anthropogenic crop was found in DHR summer diets (*Zea mays*) and two were found in KSL summer diets (*Z. mays*; and Kodo millet [*Paspalum scrobiculatum*]). Other than insects, no animal prey was found in either diet. The proportions of macronutrients in diets (i.e., realized macronutrient niches) were relatively high in carbohydrate for both study areas and seasons: DHR_summer_ 24.1P:8.7L:67.2C; KSL_summer_ 16.7P:8.2L:75.1C; DHR_autumn_ 21.1P:10.5L:68.4C; KSH_autumn_ 19.0P:11.0L:70.0C. Macronutrient niche breadth was 3.1 × greater in the DHR than KSL during summer, and 4.0 × greater in the autumn, primarily due to the higher proportion of lipid in ants and termites relative to plant foods. Within‐study area differences in niche breadth were greater during summer than autumn; in the KSH the macronutrient breadth was 1.4 × greater in summer, while in the DHR it was 1.1 × greater in summer. Similarity in dietary macronutrient proportions despite differences in foods consumed and niche breadth are suggestive of foraging to reach a preferred macronutrient balance.

## 1 INTRODUCTION

Understanding the diet of a species is necessary for unraveling the complexities of its ecology. Diet itself can be thought of as being composed of mixtures of foods (i.e., meals) consumed by an animal (Raubenheimer & Simpson, 2016). Foods, in turn, are composed of mixtures of nutrients and other non‐nutritional components which can strongly guide animal foraging behavior (Righini, 2016). The macronutrients (protein, carbohydrate, lipid), which are metabolized for essential biological processes and energy provisioning (Kohl, Coogan, & Raubenheimer, 2015), have been shown to strongly influence the foraging behavior of many species (e.g., Coogan et al., 2017; Johnson et al., 2017; Rowe, Figueira, Raubenheimer, Solon‐Biet, & Machovsky‐Capuska, 2018). Studies have demonstrated the ability of animals to select nonrandom proportions of dietary macronutrients that, in turn, optimize some aspect of their fitness and minimize the deleterious effects of confinement to an imbalanced diet (Jensen et al., 2012; Raubenheimer & Simpson, 1997). Foraging can thus be thought of as a dynamic homeostatically regulated behavior aimed at optimizing the intake of available nutrient mixtures (Guo et al., 2017; Raubenheimer & Simpson, 2018).

Traditionally, the variety (i.e., breadth) of foods consumed by a species has been used to characterize a species niche, including classification as a specialist or generalist (Hutchinson, 1957; Machovsky‐Capuska, Senior, Simpson, & Raubenheimer, 2016). Given the influence of nutrients on animal behavior and fitness, nutritional ecologists have recently started examining the nutrient compositions of species’ diets in the context of niche theory (Coogan, Raubenheimer, Stenhouse, Coops, & Nielsen, 2018; Machovsky‐Capuska, Amiot, Denuncio, Grainger, & Raubenheimer, 2018; Machovsky‐Capuska, Senior et al., 2016; Senior, Grueber, Machovsky‐Capuska, Simpson, & Raubenheimer, 2016). This multidimensional nutritional niche framework characterizes the niche of species across four functional levels: (a) the “food exploitation niche” considers the physical and ecological characteristics of foods consumed; (b) the “food composition niche” describes the variation in nutritional composition of foods consumed; (c) the “realized macronutrient niche” describes the dietary macronutrient composition of a species at some level (e.g., subpopulation, population) that allows it to persist; and (d) the “fundamental macronutrient niche” describes the full range of dietary macronutrient compositions that a species can physiologically persist on.

Omnivorous species are broadly considered generalists. For instance, the brown bear (*Ursus arctos*) consumes a wide variety of foods varying in both physical and nutritional properties, and can thus be considered a generalist in terms of both food exploitation and food composition (Coogan, Raubenheimer, Stenhouse et al., 2018). The realized macronutrient niches of brown bear populations were shown to vary widely, suggesting a broad fundamental macronutrient niche (Coogan, Raubenheimer, Stenhouse et al., 2018). Brown bears also experienced variation in dietary macronutrient composition seasonally, due to their reliance on seasonally available foods (Coogan, Raubenheimer, Stenhouse et al., 2018; Coogan, Raubenheimer, Stenhouse, & Nielsen, 2014). Under ad libitum experimental conditions, however, brown bears self‐selected a relatively high proportion of nonprotein macronutrients (lipid and carbohydrate) relative to protein across seasons (Erlenbach, Rode, Raubenheimer, & Robbins, 2014). The macronutrient preferences of the bears were also shown to maximize their mass gain, which was considered a proxy for fitness, suggesting that their dietary preferences were adaptive. The macronutrient preferences of brown bears likely play a role in food‐related conflict with humans, particularly when natural foods that satisfy their high‐lipid (e.g., hard mast) or high‐carbohydrate (e.g., soft mast) preferences are scarce (Coogan & Raubenheimer, 2016). In fact, brown bears with diets containing anthropogenic foods were found to have diets proportionally higher in carbohydrate than bears with natural diets (Coogan, Raubenheimer, Stenhouse et al., 2018).

The Asiatic black bear (*Ursus thibetanus*) is similar to the brown bear in that they have an omnivorous diet, consuming a wide range of seasonally and regionally available foods diverse in macronutrient composition (Furusaka et al., 2017; Hwang, Garshelis, & Wang, 2002; Hyugens et al., 2003; Schaller et al., 1989). The macronutrient preferences of Asiatic black bear have to date not been determined to the best of our knowledge. Furthermore, relatively little is known of their diet in Nepal. This is problematic, as Asiatic black bears have been documented frequenting both corn (*Zea mays*) and rice fields in the Annapurna Conservation Area of Nepal creating food‐related human‐wildlife conflict (Bista & Aryal, 2013). In fact, the Asiatic black bear face several threats due to human activity. The species is listed as “vulnerable” by the IUCN (Garshelis & Steinmetz, 2016), due to widespread illegal killing, trade in bear parts, and habitat loss (e.g., Ahmadzadeh et al., 2008; Escobar, Awan, & Qiao, 2015). The species is also included in Appendix I of the Convention on International Trade in Endangered Species of Wild Fauna and Flora (CITES, 2017). Thus, better understanding the diet and macronutrient niche of Asiatic black bear in Nepal will serve to help identify important natural foods, potentially problematic anthropogenic foods, and inform the conservation and management of important habitat in the country.

In this paper, we identified foods consumed by Asiatic black bear in two regions of Nepal, the Dhorpatan Hunting Reserve (DHR) and the Kailash Sacred Landscape (KSL), during the summer and autumn seasons. We used nutritional geometry (Raubenheimer, 2011) to examine the proportions of macronutrients in the foods and diets of bears in both regions, and from this the seasonal realized macronutrient niches of both subpopulations, using data from proximate analyses of samples collected in the field as well as from the literature. We predict that the macronutrient proportions of diets will be relatively high in nonprotein macronutrients (combinations of carbohydrate and lipid) compared to protein, given that previous studies of Asiatic black bear document them as consuming high proportions of both soft and hard mast during summer and autumn. This prediction is also in keeping with diet studies of brown bear (Coogan et al., 2014; López‐Alfaro, Coogan, Robbins, Fortin, & Nielsen, 2015) and American black bear (*Ursus americanus*; Beeman & Pelton, 1980). In addition to providing regional knowledge of Asiatic black bear nutritional ecology, this research will contribute to the nascent comparative nutritional ecology literature.

## 2 MATERIALS AND METHODS

### 2.1 Study areas

**Figure 1:**
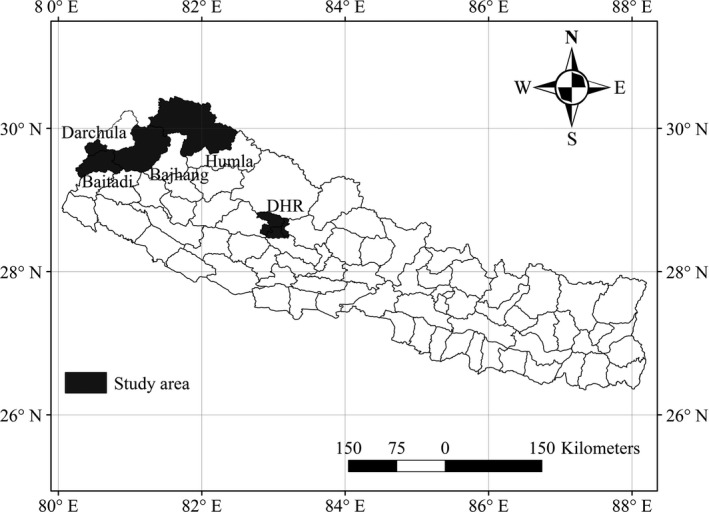
Map of Nepal showing the two study areas in which scats of Asiatic black bear were collected to determine summer (June–July) and autumn (October–November) diets: (a) Dhorpatan Hunting Reserve (DHR); and (b) the Kailash Sacred Landscape (KSL), which includes the Baitadi, Bajhang, Darchula, and Humla districts

The DHR covers ~1,325 km^2^ and is situated in western Nepal within the Rukum, Myagdi, and Baglung districts in the Dhaulagiri Himal range (Figure 1). Elevation (derived from a digital elevation model) ranges from 1,893 to 7,308 m. The DHR provides habitat for many mammalian species, including barking deer (*Muntiacus muntjak*), rhesus macaque (*Macaca mulatta*), wolf (*Canis lupus*), red panda (*Ailurus fulgens*), wild boar (*Sus scrofa*), common leopard (*Panthera pardus*), Himalayan goral (*Naemorhedus goral*), and Himalayan serow (*Capricornis thar*; DNPWC, 2017; Panthi, Khanal, Acharya, Aryal, & Srivathsa, 2017). The region is well known for trophy hunting of blue sheep (*Pseudois nayaur*) and Himalayan tahr (*Hemitragus jemlahicus*; Aryal et al., 2015). The DHR also provides habitat for *Ophiocordyceps sinensis*, a traditional medicinal fungus commonly known as “Himalayan Viagra” that is harvested by humans (Thapa et al., 2014).

The KSL is a transboundary high mountain region in the Himalayas shared between China, India, and Nepal. Our second study area (11,015 km^2^) was in the Nepalese part of the KSL, which includes the Baitadi, Bajhang, Darchula (with the exception of the Api Nampa Conservation Area), and Humla districts (Figure 1). Elevation ranges from 374 to 7,041 m. The KSL is rich in biodiversity, as it is home to a number of endemic and threatened species including the elusive snow leopard (*Panthera uncia*; ICIMOD, 2009; Uddin et al., 2015). Forests ecosystems are being degraded in this landscape due to high dependency on forest resources for livelihoods and conversion to cropland (Uddin et al., 2015).

### 2.2 Scat collection and diet estimation

We collected fresh black bear scats from the KSL and DHR during June and July (summer) and October to November (autumn) of 2016 following the procedure used by Panthi, Aryal, Raubenheimer, Lord, and Adhikari (2012) Panthi, Coogan, Aryal, and Raubenheimer (2015) to determine the diet of red panda (*A. fulgens*) in the DHR. That is, potential and known habitat of Asiatic black bear was identified based on local knowledge, observations, or signs (e.g., paw prints, scats, bed sites). Man‐made and game trails in black bear habitat were walked to identify and collect scats. Scats were identified by the first author (SP) and experienced field assistants. We collected samples within the elevation range of approximately 1,800–3,500 m for both study areas. Black bears in central Nepal have been shown to prefer habitats in the range of 1,600–3,200 m (Bista & Aryal, 2013), while the IUCN Redlist gives the species an elevation range of 0–4,300 m (Garshelis & Steinmetz, 2016). Other bear species (e.g., Himalayan brown bear) are generally not known to inhabit the areas where scats were sampled, thus simplifying scat identification.

We used microhistological fecal analysis (Holechek & Gross, 1982) to determine the diet composition (% relative frequency [RF%]) of black bear following Panthi et al. (2012), Panthi et al. (2015). Reference samples (plants and insects) were collected from the study areas for microhistology. For each sample group of scats, 1 slide was prepared for examination and 20 fragments were randomly selected from each slide to compare with reference samples.

To examine dietary macronutrient proportions, we collected plants identified as part of the diet of the Asiatic black bear for proximate nutritional analysis during the summer (June) and autumn (November) of 2016. For each plant, we collected samples from a minimum of five different sites. Samples were air dried and kept in plastic bags for transport to the laboratory. Samples of the same species were combined from all sites in each study area for proximate analysis of the composite samples at the Nepal Environmental and Scientific Services laboratory (Kathmandu), and the Nepal Agricultural Research Council, Animal Nutrition Division (Khumaltar, Lalitpur). Proximate analysis included estimation of crude protein (micro‐Kjeldahl method), ether extract (i.e., crude fat; Soxhlet extraction), crude fiber (digestion method), moisture (gravimetric), and ash (gravimetric). Available carbohydrate was estimated by subtraction. We did not collect ants or termites for proximate analysis; thus, we obtained representative average estimates from the literature (Rumpold & Schlüter, 2013). We also used estimates for some plant species in the DHR from Panthi et al. (2015).

The macronutrient proportions of foods consumed by bears in each study area and season were estimated from the proximate analyses by first converting each macronutrient to units of metabolizable energy in kcal/g (Coogan et al., 2014) using standardized conversion factors (i.e., protein and carbohydrate 4 kcal/g; and lipid 9 kcal/g; Merrill & Watt, 1973). Each macronutrient was then expressed as a percentage of total macronutrient‐derived metabolizable energy to examine the proportion of macronutrients in individual foods. The macronutrient proportions of seasonal and study area‐specific diets were calculated by weighting individual food macronutrient proportions by their RF% in the diet (after correcting for unidentified material) and summing them. These diet estimates serve to represent the realized macronutrient niches of bears in each study area for each season (Coogan, Raubenheimer, Stenhouse et al., 2018; Coogan, Raubenheimer, Zantis, & Machovsky‐Capuska, 2018b). The dietary macronutrient breadth for each study area and season was assessed visually within a right‐angled mixture triangle plot (Raubenheimer, 2011), by constructing convex hulls around dietary food points using the chull function in the R (v. 3.4.4) package {grDevices} (R Core Team, 2018). The relative differences in dietary macronutrient breadth between study areas and seasons were quantified by calculating the area of convex hull polygons using the areapl function in the R package {splancs} (Rowlingson & Diggle, 2017), and expressed as a relative effect size.

## 3 RESULTS

### 3.1 Diet estimates

We collected a total of 209 scats: 41 summer, and 32 autumn, samples from the DHR; and 77 summer, and 59 autumn, samples from the KSL. Bamboo (*Arundinaria *spp.) had the highest RF% of all food items for both study areas and seasons (Tables 1 and 2). In DHR summer diets (Table 1), we identified a total of nine plant species as well as ants (Formicidae). One species each of hard mast, soft mast, fern, and anthropogenic crop (*Z. mays*) were identified in the DHR summer diet, but not in the autumn diet. Six species of plants were identified in autumn DHR diets (Table 1), as well as both ants and termites (Blattodea). *Rhododendron* spp. had the second highest RF% in autumn and was not found in summer diets. No hard mast, soft mast, or maize was found in the autumn DHR diet.

**Table 1:** Summer and autumn diet (percent relative frequency [RF %]) of Asiatic black bear in the Dhorpatan Hunting Reserve (DHR), Nepal

Food item	Category	Summer (RF %)	Autumn (RF %)
Yeiselu (*Rubus ellipticus*)	Buds and twigs	3.2	7.5
Nigalo (*Arundinaria* spp.)	Bamboo	34.6	30.2
Chutro (*Berberis aristata*)	Buds and twigs	7.9	12.5
Jhayu (Lichen)	Lichen	5.6	13.3
Kharsu leaf (*Quercus semicarpifolia*)	Leaf	–	6.7
Guransh (*Rhododendron *spp.)	Leaf	–	17.0
Ants (Formicidae)	Insect	3.3	4.3
Termites (Blattodea)	Insect	–	3.1
Paskate (unknown)	Leaf	3.1	–
Kharsu seed (*Quercus semicarpifolia*)	Hard mast	5.6	–
Banko seed (*Arisaema tortuosum var. curvatum)*	Soft mast	11.8	–
Maize seed (*Zea mays*)	Crop	12.8	–
Fern (*Matteuccia struthiopteris*)	Fern	10.5	–
Unidentified	–	1.6	5.4
Total	–	100	100

**Table 2:** Summer and autumn diet (percent relative frequency [RF %]) of Asiatic black bear in the Kailash Sacred Landscape (KSL), Nepal

Food item	Category	Summer (RF %)	Autumn (RF %)
Chutro (*Berberis aristata*)	Buds and twigs	2.3	3.3
Nigalo (*Arundinaria* spp.)	Bamboo	24.2	21.5
Khanyu Seed (*Ficus semicordata*)	Soft mast	3.4	7.2
Ghamari (unknown sp.)	Leaf	5.2	7.9
Banjh (*Quercus incana*)	Hard mast	7.1	8.6
Fern (*Matteuccia struthiopteris*)	Fern	9.2	15.9
Yeiselu (*Rubus ellipticus*)	Buds and twigs	3.4	7.8
Guransh (*Rhododendron* spp.)	Leaf	–	13
Jhayu (Lichen)	Lichen	–	9.4
Banko Seed (*Arisaema tortuosum var. curvatum*)	Soft mast	9.5	–
Kodo millet (*Paspalum scrobiculatum*)	Crop	9.8	–
Wild pear seed (*Pyrus pyraster*)	Soft mast	13.2	–
Maize seed (*Zea mays*)	Crop	10.2	–
Unidentified	–	2.5	5.4
Total	–	100	100

In KSL summer diets (Table 2), we identified 11 species of plants, including hard mast (*n* = 1), soft mast (*n* = 3), and two anthropogenic crops (*Z. maize*, and Kodo millet [*Paspalum scrobiculatum*]). In KSL autumn diets, one species of soft mast (*Ficus semicordata*) and hard mast (*Quercus incana*) continued to be found in the diet, with no evidence of anthropogenic crops consumed. No insects were found in the KSL diet, and bamboo had a lower RF% for both seasons compared to the DHR. Other than insects in the DHR diet, no animal prey was found in scats in either study area.

### 3.2 Macronutrient proportions of foods and diets

**Figure 2:**
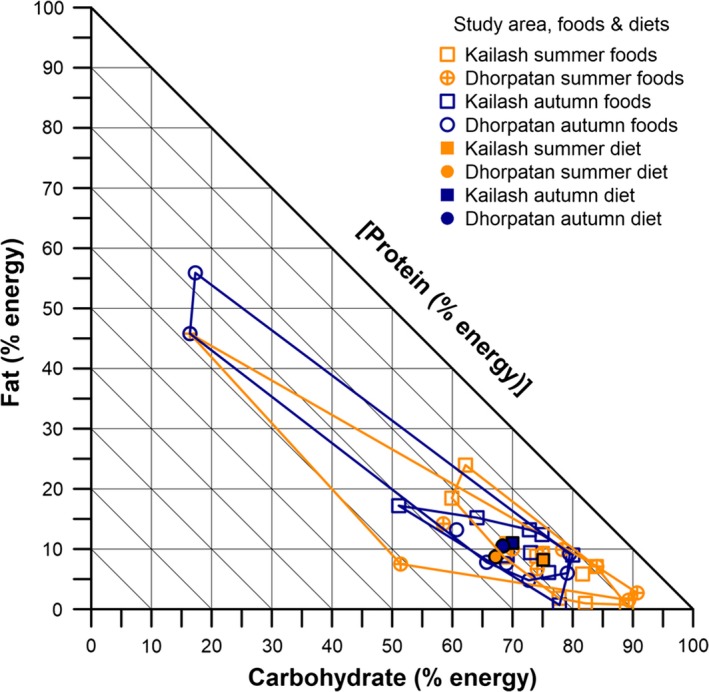
Right‐angled mixture triangle depicting the macronutrient proportions in the foods and diets of Asiatic black bear in two separate seasons and study areas (Kailash Sacred Landscape; Dhorpatan Hunting Reserve) in Nepal. Convex hull polygons depict dietary macronutrient breadth of food items for each season and study area. Solid symbols depict the estimated proportional macronutrient composition of the diet consumed by the bears based on the relative frequency of food items in scats. Diet points can serve to represent realized macronutrient niches

Proximate nutritional analysis of composite samples showed that some foods in the KSL (e.g., *Arundinaria* spp., *Berberis aristata*) had noticeably lower crude protein, and higher crude fiber, content compared to plants from the DHR (Table 3). Overall, the dietary proportions of macronutrients were relatively high in carbohydrate in both study areas and seasons (Figure 2). Macronutrient breadth of food items (i.e., volume of convex hull polygons) was 3.1 × greater in the DHR than KSL during summer, and 4.0 × greater in the autumn. This was despite the KSL diets having one more total food items than DHR diets for each season. The larger macronutrient breadth in the DHR was due primarily to the presence of both ants and termites in the diet, which had greater proportional lipid, and lower carbohydrate, content relative to the plant foods—ants (protein % [P]:lipid % [L]:carbohydrate %[C] = 37.7P:45.8L:16.4C) and termites (26.8P:55.9L:17.3C). The proportionally high protein estimate of bamboo (41.0P) in the DHR, lower protein estimates of bamboo (22.7P) and other species in the KSL, and greater consumption of high‐carbohydrate crops and mast in the KSL resulted in diets proportionally higher in protein and lower in carbohydrate in the DHR (24.1P:8.7L:67.2C) during summer compared to the KSL (16.7P:8.2L:75.1C). Autumn diets, however, were more similar between study areas (DHR, 21.1P:10.5L:68.4C; KSH, 19.0P:11.0L:70.0C) despite differences in the types and macronutrient proportions of foods consumed. Within‐study area differences in seasonal macronutrient breadth were less pronounced, but greater during summer than autumn; in the KSH, the macronutrient breadth was 1.4 × greater in summer than autumn, while in the DHR it was 1.1 × greater in summer.

**Table 3:** Proximate estimates of Asiatic black bear plant foods in two study areas of Nepal (DHR = Dhorpatan Hunting Reserve; KSL = Kailash Sacred Landscape) during two seasons

Food item	Ash (%)	CP (%)	EE (%)	Moisture (%)	CF (%)	AC (%)
DHR, Summer (June–July)						
Yeiselu (*Rubus ellipticus*)	8.96	12.12	2.71	11.00	22.58	42.62
Nigalo (*Arundinaria *spp.)	22.00	20.31	1.66	8.45	22.10	25.48
Chutro (*Berberis aristata*)^a^	6.50	18.66	4.33	9.5	21.01	40.00
Jhayu (Lichen)^a^	6.20	8.00	2.96	10.90	19.01	52.93
Paskate	9.76	11.23	1.73	9.91	23.95	43.41
Kharsu seed (*Quercus semicarpifolia*)	7.98	4.98	0.90	7.98	9.89	68.28
Banko seed (*Arisaema tortuosum var. curvatum*)	8.78	7.12	0.50	7.70	6.90	69.00
Maize seed (*Zea mays*)	3.65	8.05	2.82	7.58	2.44	75.46
DHR, Autumn (October–November)						
Yeiselu (*Rubus ellipticus*)	9.34	12.17	1.75	12.65	28.17	35.92
Nigalo (*Arundinaria *spp.)^a^	9.10	12.44	1.18	12.04	25.06	40.18
Chutro (*Berberis aristata*)^a^	5.48	17.86	2.33	9.97	20.13	44.23
Jhayu (Lichen)^a^	5.72	8.94	1.61	14.06	22.24	47.43
Kharsu leaf (*Quercus semicarpifolia*)	9.97	15.27	3.41	9.21	26.73	35.40
Lali Guransh (*Rohododendron *spp.)	10.23	7.82	2.85	12.13	11.72	55.25
KSL, Summer (June–July)						
Chutro (*Berberis aristata*)	3.56	9.21	2.23	8.90	44.33	31.77
Nigalo (*Arundinaria* spp.)	10.54	8.24	1.40	9.08	45.87	24.87
Khanyu seed (*Ficus semicordata*)	12.87	6.99	1.45	8.98	24.16	45.55
Ghamari	10.84	16.03	6.09	9.95	12.66	44.43
Banjh (*Quercus incana*)	8.98	9.32	0.37	7.71	38.23	35.40
Fern (*Matteuccia struthiopteris*)	6.97	8.00	2.09	8.70	36.17	38.07
Yeiselu (*Rubus ellipticus*)	8.56	10.07	2.31	10.13	25.43	43.50
Banko seed (*Arisaema tortuosum var. curvatum*)	9.12	11.59	8.90	8.97	9.40	52.01
Millet (*Paspalum scrobiculatum*)	9.21	7.02	0.18	14.32	35.18	34.08
Wild pear seed (*Pyrus pyraster*)	12.15	3.69	0.12	10.93	41.34	31.78
Maize seed (*Zea mays*)	3.65	8.05	2.82	7.90	2.53	75.05
KSL, Autumn (October–November)						
Chutro (*Berberis aristata*)	6.87	11.64	2.76	9.93	20.51	48.29
Nigalo (*Arundinaria *spp.)	9.45	9.65	3.15	13.10	34.72	29.93
Khanyu seed (*Ficus semicordata*)	11.98	10.58	0.15	8.81	30.28	38.19
Ghamari	11.87	7.63	3.32	10.78	21.44	44.96
Banjh (*Quercus incana*)	9.87	14.17	3.41	8.19	41.52	22.83
Fern (*Matteuccia struthiopteris*)	7.95	11.11	1.99	8.89	35.25	34.82
Yeiselu (*Rubus ellipticus*)	7.09	11.40	1.72	11.06	20.52	48.21
Guransh (*Rhododendron* spp.)	10.23	7.82	2.85	12.13	10.19	56.78
Jhayu (Lichen)	6.73	7.38	3.09	13.35	31.19	38.26

Note

Ants and termites found in DHR diets were not analyzed.

AC: available carbohydrates; CF: crude fiber; CP: crude protein; EE: ether extract.

a Estimates from Panthi et al. (2015) for plants sampled in the DHR from June–July 2013 to November 2012–February 2013.

Because of relatively small sample sizes, we estimated the macronutrient balance of the overall diet from the 209 total scats, which was 20.2P:9.6L:70.2C. The mean seasonal and study area diets are presented in Table 4. Within study areas, coefficient of variation (CV) for lipid was relatively high, indicating greater standardized variation in the mean proportion of lipid consumed between seasons in the same study area, than between study areas during the same season. There was also a relatively higher CV for the mean proportion of protein consumed during summer, suggesting greater standardized variation in protein intake between study areas during the summer.

**Table 4:** Mean seasonal (both study areas) and mean study area (both seasons) diet proportions of metabolizable energy from protein, lipid, and carbohydrate, including standard deviation (*SD*) and coefficient of variation (CV)

Season or study area	Protein	Lipid	Carbohydrate
Summer (*n* = 118)			
Mean	20.4	8.4	71.2
*SD*	5.3	0.3	5.6
CV	0.26	0.04	0.08
Autumn (*n* = 91)			
Mean	20.1	10.7	69.2
*SD*	1.5	0.3	1.2
CV	0.07	0.03	0.02
KSL (*n* = 136)			
Mean	17.8	9.6	72.6
*SD*	1.6	2.0	3.6
CV	0.09	0.21	0.05
DHR (*n* = 73)			
Mean	22.6	9.6	67.8
*SD*	2.14	1.31	0.83
CV	0.09	0.14	0.01

Note

DHR: Dhorpatan Hunting Reserve; KSL: Kailash Sacred Landscape.

## 4 DISCUSSION

The Asiatic black bear can be considered a generalist in terms of the food exploitation niche and macronutrient composition niche based on a priori knowledge. We found that the realized macronutrient niches of Asiatic black bear in both Nepalese study areas were similar, despite differences in macronutrient niche breadth, suggesting that they were regulating their diet toward a shared and preferred proportion of dietary macronutrients. The realized niches of Asiatic black bears in our study were similar to those of global brown bear populations during autumn (Coogan, Raubenheimer, Stenhouse et al., 2018). Likewise, the proportion of macronutrients in the black bear's diets were similar to those self‐selected by captive brown bears, which maintained an average ratio of 17% protein to an 83% mixture of carbohydrates and lipids (i.e., nonprotein macronutrients; Erlenbach et al., 2014)—similarity in macronutrient preferences among species of bears is a possibility, because brown bear, American black bear, and giant panda (*Ailuropoda melanoleuca*) were shown to have similar digestive efficiencies (Pritchard & Robbins, 1990). This ratio of macronutrients was found to maximize mass gain per unit energy intake in brown bear and coincides ecologically with the nutritional compositions of foods available in the hyperphagic prehibernation period in which brown bears attempt to acquire sufficient body mass for hibernation (López‐Alfaro, Robbins, Zedrosser, & Nielsen, 2013). Hibernation varies for Asiatic black bears depending upon location; those in temperate areas have been shown to hibernate, while those in tropical or subtropical areas do not hibernate (Hwang & Garshelis, 2007; Reid, Jiang, Teng, Qin, & Hu, 1991; Seryodkin et al., 2003). Pregnant females are the exception, as they den to produce altricial young (Hwang & Garshelis, 2007). The hibernation habits of Asiatic black bear in Nepal have not to our knowledge been documented, but it is likely that they move to lower elevations in the winter without hibernating. Regardless, the pattern of eating high‐fat or high‐carbohydrate mast during this period tends to occur whether the Asiatic black bear hibernates or not (Hwang & Garshelis, 2007).

Studies of Asiatic black bear report seasonal shifts in diet from predominantly graminoids and forbs in spring, soft mast in summer, and hard mast in fall (Schaller et al., 1989; Huygens et al., 2003). In fact, the Asiatic black bear is considered to be ecologically similar to the American black bear in part due to their dietary similarities (Hwang & Garshelis, 2007). In this paper, we show that bamboo had the highest RF% in both summer and autumn, making up approximately 1/3 to 1/5 of the RF% of food items consumed in both study areas, suggesting its importance in the diet of bears in Nepal. Bamboo was also found in the summer and autumn diets of bears in other countries, such as Sichuan, China (Reid et al., 1991), and to a lesser extent in the northern Japanese Alps (Hyugens et al., 2003). Based on our study, no animal prey other than insects were found in the diet, which is consistent with other reports of animal prey (e.g., mammals, fish, birds, insects) contributing a relatively small proportion to Asiatic black bear diets (Schaller et al., 1989; Hwang et al., 2002; Hyugens et al., 2003).

We found evidence that bears in both study areas consumed anthropogenic crops during the summer, which has important conservation implications. Maize was consumed in both study areas, while Kodo millet was also consumed in the KSL. Both of these crop foods have a high proportion of carbohydrate energy (~82–84°C), which further demonstrates a link between high‐carbohydrate foods and bear‐human conflict (Coogan & Raubenheimer, 2016), yet in this case for Asiatic black bear. Furthermore, diets of bears in the KSL, which had approximately 1.6 × higher RF% of crop foods in summer compared to the DHR (12.8 RF% in DHR vs. 20.0 RF% in KSL), were also proportionally higher in carbohydrate and lower in protein, which is consistent with a global review of brown bear diets (Coogan, Raubenheimer, Stenhouse et al., 2018). Anthropogenic crop depredation has been documented throughout the range of Asian black bear (Can, D'Cruze, Garshelis, Beecham, & Macdonald, 2014; Hwang et al., 2002; Hyugens, Manen, Marotello, Hayashi, & Ishida, 2004; Reid et al., 1991; Sathyakumar & Viswanath, 2003) and has been linked to natural limitations in the availability of both hard and soft mast (Honda, 2013). An ethological perspective on bear foraging behavior, specifically, nutrient‐specific nutritional preferences (e.g., Simpson, Sibly, Lee, Behmer, & Raubenheimer, 2004), also implicates the nutritional composition of depredated foods as key drivers of such conflict behavior (Coogan & Raubenheimer, 2016)—depredated foods can have similar nutritional properties to preferred natural foods which may be limiting. Furthermore, anthropogenic food might also be more reliable, easily obtained, and spatially concentrated than natural food. Retaliatory killing of bears for crop and livestock depredation is a threat to their conservation (Can et al., 2014; Hyugens et al., 2004; Wang, Lassoie, & Curtis, 2006), and livestock depredation has also been documented in other countries (e.g., Sathyakumar, 2001; Sangay & Vernes, 2008); however, we found no evidence of livestock in the diets of bears in this study.

It is possible that the differences in the dietary balance of carbohydrate and protein in summer diets between subpopulations may lead to body composition differences between bears. In laboratory studies of mice models, for example, higher proportions of dietary carbohydrate relative to protein tend to result in a higher body fat composition, while higher proportions of protein tend to be associated with greater lean mass (e.g., Solon‐Biet et al., 2014). The proportion of lipid in the diets of bears was relatively consistent and lower than the other macronutrients.

We found intraspecific variation in plant nutritional characteristics between sites. The source of this intraspecific variation between study areas is unknown, but could reflect differences in site‐level characteristics affecting plant nutritional composition and genetic factors. The nutritional differences observed may also reflect the habitat degradation occurring the KSL (Uddin et al., 2015). Such variation is not unprecedented, as the nutritional composition of plant samples of the same species can sometimes vary markedly across an animal's range (Rothman, Chapman, & Soest, 2012). However, because the samples analyzed were composites of multiple plants across sites, we assumed that nutritional estimates were representative of the study area in general.

In conclusion, we present seasonal dietary and nutritional information of the Asiatic black bear in Nepal, which furthers our understanding of their behavior and nutritional ecology. Conducting work on the nutritional ecology of free‐ranging carnivores is a challenging task (Machovsky‐Capuska, Coogan, Simpson, & Raubenheimer, 2016), and as such we suggest a few recommendations for increasing our knowledge of Asiatic black bears in the region, and as a species, including characterization of annual diet, identifying the nutritional preferences of the bears, sample collection or biologging of known individuals, expanding the geographic range of studies, elucidating the hibernation habits of Nepalese bears, developing fecal correction factors, and further understanding the nutritional factors related to human‐bear conflict. Although challenging, such efforts will go a long way to furthering our understanding of this imperiled omnivore.

## CONFLICT OF INTEREST

None declared.

## AUTHOR CONTRIBUTIONS

SP and AA conceived the project and designed the study. SP collected the data, analyzed scat samples, and estimated diets. SCPC interpreted the data, analyzed macronutrient proportions, and led the writing of the paper. All authors critically reviewed the paper.

## Data Availability

Data accessibility: Dryad 10.5061/dryad.81kk648.
